# Radiation therapy improves survival in rectal small cell cancer - Analysis of Surveillance Epidemiology and End Results (SEER) data

**DOI:** 10.1186/s13014-015-0411-y

**Published:** 2015-04-24

**Authors:** Aram S Modrek, Howard C Hsu, Cynthia G Leichman, Kevin L Du

**Affiliations:** Medical Scientist Training Program, New York, USA; Department of Radiation Oncology, New York, USA; Division of Hematology and Medical Oncology, Department of Medicine, New York University School of Medicine, New York, USA

## Abstract

**Background:**

Small cell carcinoma of the rectum is a rare neoplasm with scant literature to guide treatment. We used the Surveillance Epidemiology and End Results (SEER) database to investigate the role of radiation therapy in the treatment of this cancer.

**Methods:**

The SEER database (National Cancer Institute) was queried for locoregional cases of small cell rectal cancer. Years of diagnosis were limited to 1988–2010 (most recent available) to reduce variability in staging criteria or longitudinal changes in surgery and radiation techniques. Two month conditional survival was applied to minimize bias by excluding patients who did not survive long enough to receive cancer-directed therapy. Patient demographics between the RT and No_RT groups were compared using Pearson Chi-Square tests. Overall survival was compared between patients who received radiotherapy (RT, n = 43) and those who did not (No_RT, n = 28) using the Kaplan-Meier method. Multivariate Cox proportional hazards model was used to evaluate important covariates.

**Results:**

Median survival was significantly longer for patients who received radiation compared to those who were not treated with radiation; 26 mo vs. 8 mo, respectively (log-rank P = 0.009). We also noted a higher 1-year overall survival rate for those who received radiation (71.1% vs. 37.8%). Unadjusted hazard ratio for death (HR) was 0.495 with the use of radiation (95% CI 0.286-0.858). Among surgery, radiotherapy, sex and age at diagnosis, radiation therapy was the only significant factor for overall survival with a multivariate HR for death of 0.393 (95% CI 0.206-0.750, P = 0.005).

**Conclusions:**

Using SEER data, we have identified a significant survival advantage with the use of radiation therapy in the setting of rectal small cell carcinoma. Limitations of the SEER data apply to this study, particularly the lack of information on chemotherapy usage. Our findings strongly support the use of radiation therapy for patients with locoregional small cell rectal cancer.

## Background

Neuroendocrine neoplasias (NEN) arise from neural and endocrine cell types and can occur all throughout the body. NEN are broadly grouped into pulmonary and extrapulmonary primary sites and encompass a heterogeneous class of malignancies that vary widely in their clinical presentations and characteristics. The number of different iterations of NEN that arise as a function of primary site, grade and clinical presentation compounded by their relative infrequency has made clinical trial execution difficult and slowed the pace at which outcomes can be improved for the many subtypes [1-3]. Grade I/II NEN are by definition neuroendocrine tumors (NET). Some of the literature use the term NET to refer more broadly to NEN, which encompass all grades of neuroendocrine neoplasms. The more aggressive and poorly differentiated grade III neuroendrocrine carcinomas (NEC) are further subdivided into small cell carcinomas (SCC) and large cell carcinomas (LCC).

Surveillance, Epidemiology and End Results (SEER) studies examining NEN epidemiology revealed an incidence rate of 5.25 and 5.76 per 100,000 for the 2000–2004 and 2003–2007 periods, respectively [4,5]. Both of these studies note a greater than four-fold increase in the incidence of NEN for the data available since 1973, which the authors of the studies attribute to improved diagnostic capabilities and greater disease awareness. Patients with well or moderately differentiated diagnoses had median survival times of 124 and 64 months, respectively, while those diagnosed with poorly differentiated tumors (such as SCC or LCC) had a 10 month median survival time [5]. The stark difference between mortality rates of low and high grade NEN highlights the importance of establishing more accurate and consistent grading criteria to guide future treatments, distinguish between indolent and aggressive subtypes and provide meaningful prognostic information.

Extrapulmonary GI NEN as a group make up 61% of all NEN diagnoses, with the rectum being the most common site (17.7%), followed by the small intestines (17.3%) and colon (10.1%) [4]. Amongst the more aggressive and rare GI SCC, the most common site of incidence is the esophagus (53%) followed by the colon (13%), stomach (11%), gall bladder (8.4%) and rectum (7.3%) [4,6]. SCC of the rectum are aggressive and rare tumors compromising less than 1% of all GI neoplasms, with reported median survival ranging from 7 to 11 months and estimated five year survival rates of 8-15% [5,7-12]. Due to its low incidence, there is a paucity of literature to guide treatment and we are currently limited to case reports and small retrospective studies. Treatments for extrapulmonary GI SCC have been guided by extrapolating from treatment modalities used for small cell lung cancer [2,9,13]. Nonetheless, in the published literature there was tremendous variation in treatment approaches, outcomes and follow-up information. Given the scarcity of literature and rare incidence of rectal SCC, we performed a SEER database analysis to understand the potential role of radiotherapy in the treatment of this malignancy.

## Methods

The SEER database (National Cancer Institute) was queried with SEER*Stat8.1.2 software for rectal small cell cancer cases. In order to exclude patients with distant metastatic disease, we restricted the search to locoregional cases by using the “SEER Historic Stage A” variable. Years of diagnosis were restricted to 1988–2010, in order to reduce bias or error due to variability in staging/grading criteria and longitudinal changes in chemotherapy, surgery or radiation treatment techniques. Radiation in the RT group was limited to external beam radiation therapy for all cases. Surgical procedures were grouped based on the extent of resection. A two month conditional survival was applied to minimize bias by excluding patients who did not survive long enough to receive cancer-directed therapy. Overall survival was compared between patients who received radiation (RT) and those who did not (No_RT) using the Kaplan-Meier method. Multivariate Cox proportional hazards statistical modeling was used to evaluate important covariates, with AJCC Stage as a stratification variable. Patient demographics between the RT and No_RT groups were compared using Pearson Chi-Square tests.

## Results

The SEER database included 71 patients that fit our study criteria. This patient cohort had 43 patients treated with radiation (RT group) and 28 patients without radiation (No_RT group). There were no significant difference between the RT and No_RT groups when considering demographic factors such as sex, race and stage **(**Table 1**)**. There were also no statistical differences between patient location, marital status and year of diagnosis (data not shown). We did note a statistically significant difference in the sequence number in which rectal SCC was diagnosed; patients in the No_RT arm had a significant portion of rectal SCC diagnoses following another malignancy. Between the two arms of the study, the RT group received surgery 32.6% of the time, while the No_RT group received surgery 50% of the time, but this was not statistically significant with a p-value of 0.142. Chemotherapy regimen was not available through the SEER database.

**Table 1:** **Patient demographics**

		**No_RT**		**RT**		**Pearson chi-square**
		**N**	**%**	**N**	**%**	**P**
	Totals (n=71)	28		43		
Sex	Male	16	57.10%	24	55.80%	0.912
	Female	12	42.90%	19	44.20%	
Race	White	25	89.30%	40	93.00%	0.458
	Black	3	10.70%	2	4.70%	
	Other	0	0.00%	1	2.30%	
Stage	0	1	3.60%	1	2.30%	0.584
	I	8	28.60%	14	32.60%	
	II	7	25.00%	9	20.90%	
	III	9	32.10%	18	41.90%	
	Unknown	3	10.70%	1	2.30%	
Cancer diagnosis sequence number	One primary only	18	64.30%	39	90.70%	0.013
	1st of 2 or more	0	0.00%	1	2.30%	
	2nd of 2 or more	9	32.10%	2	4.70%	
	3rd of 3 or more	0	0.00%	1	2.30%	
	5th of 5 or more	1	3.60%	0	0.00%	
Radiation	None	27	96.40%	0	0.00%	N/A
	Beam radiation	0	0.00%	43	100.00%	
	Recommended, unknown if administered	1	3.60%	0	0.00%	
Surgery	None/Unknown	14	50.00%	29	67.40%	0.142
	Surgery given	14	50.00%	14	32.60%	
Radiation sequence with surgery	No radiation and/or cancer-directed surgery	28	100.00%	29	67.40%	N/A
	Radiation prior to surgery	0	N/A	2	4.70%	
	Radiation after surgery	0	N/A	12	27.90%	
Surgery Groups	No/Unknown	14	50.00%	29	67.40%	0.34
	Local surgery	7	25.00%	7	16.30%	
	Extended surgery	7	25.00%	7	16.30%	
Reason no cancer-directed surgery	Surgery performed	14	50.00%	14	32.60%	0.226
	Not recommended	10	35.70%	23	53.50%	
	Not recommended, contraindicated due to other conditions	0	0.00%	2	4.70%	
	Recommended but not performed, unknown reason	4	14.30%	2	4.70%	
	Recommended but not performed, patient refused	0	0.00%	1	2.30%	
	Recommended, unknown if performed	0	0.00%	1	2.30%	

The radiotherapy (RT) and no radiotherapy (No_RT) arms of the study were stratified based on sex, age, diagnostic sequence and treatments received. The two groups were subject to Pearson’s Chi-square statistical analysis. For the surgical categories: “No/Unknown” includes codes for: No surgery, incisional biopsy, bypass surgery only, surgery of regional site without primary site, unknown if surgery done. “Local surgery” includes codes for: local tumor excision, anterior/posterior resection, wedge or segmental resection, partial proctectomy, surgery NOS, polypectomy, excisional biopsy. “Extended surgery” includes codes for: Pull through with coloanal anastamosis, APR complete proctectomy, surgeries with partial or total removal of other organs.

**Figure 1:**
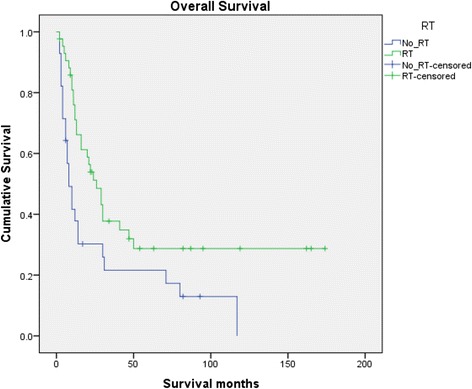
Kaplan-Meier plot of rectal small cell carcinoma cases treated with or without radiation. Rectal small cell carcinoma cases stratified by radiation treatment from the SEER database were subject to Kaplan-Meier analysis for retrospective survival benefit analysis. The no radiation therapy (No_RT) arm consisted of 28 cases. The group that received radiation therapy (RT) consisted of 43 cases.

Overall survival between the two groups differed significantly, with median survival of 26 months for the RT group versus 8 months for the No_RT group (P = 0.009) **(**Figure 1**)**. Patients who were treated with radiation had a 1 year overall survival rate of 71.1% compared to 37.8% for patients who were not treated with radiation. The unadjusted hazard ratio (HR) for death for patients that received radiation was 0.495 (95% CI 0.286-0.858). To understand how age at diagnosis, sex, surgery, and radiation affect survival, we performed univariate and multivariate cox proportional hazards modeling using AJCC stage as a stratification variable **(**Table 2**)**. Radiation therapy was the only significant factor for overall survival, with a multivariate HR for death of 0.393 (95% CI 0.206-0.750, P = 0.005). We did not observe a significant survival benefit from the use of surgery.

**Table 2:** **Cox Proportional Hazards Model with AJCC Stage as stratification variable**

**Variable**	**Level**	**No_RT N (%)**	**RT N (%)**	**Univariate HR**	**95% CI**	**P**	**Multivariate HR**	**95% CI**	**P**
Radiation	No_RT	28 (100%)	N/A	Referent			Referent		
	RT	N/A	43 (100%)	0.43	0.24 - 0.77	0.01	0.393	0.21 - 0.75	0.01
Surgery	No_Surgery	14 (32.6%)	29 (67.4%)	Referent			Referent		
	Surgery	14 (50.0%)	14 (50.0%)	0.80	0.43 - 1.49	0.47	0.53	0.26 - 1.08	0.08
Sex	Male	16 (40.0%)	24 (60.0%)	Referent			Referent		
	Female	12 (38.7%)	19 (61.3%)	0.91	0.49 - 1.70	0.76	0.90	0.47 - 1.74	0.76
**Variable**	**Level**	**No_RT Mean (SD)**	**RT Mean (SD)**	**Univariate HR**	**95% CI**	**P**	**Multivariate HR**	**95% CI**	**P**
Age at diagnosis	Continuous per year	62.4 (16.6)	58.9 (14.6)	1.02	1.0 - 1.04	0.14	1.01	0.98 - 1.03	0.32

Patients who received RT or no RT were subject to univariate and multivariate cox proportional hazards modeling (radiation, surgery, sex and age of diagnosis) with AJCC stage stratification.

## Discussion

Rectal small cell carcinoma is an aggressive and rare cancer with no standard treatment protocol. In the literature, and consistent with our own SEER analysis, the overall median survival for rectal SCC cases has ranged from 7 to 11 months [5,7-12]. Small cell lung carcinoma (SCLC) has primarily been treated with platinum based chemotherapies in conjunction with thoracic radiotherapy, and in some cases, prophylactic cranial irradiation [13-17]. However, SCLC treatments and outcomes may only go so far in translating to extrapulmonary SCC treatment. Platinum based chemotherapeutic approaches similar to those used in lung SCC have been the mainstay of treatment for many reported cases of extrapulmonary SCC [17-23]. Surgery has been used for localized disease in combination with chemotherapy or radiotherapy, but strong correlative benefits have not been established [2,9,13,23,24]. Radiotherapy used to treat extrapulmonary SCCs, with or without concurrent chemotherapy, has been reported to result in a clinical response in many cases [6,13,24-28]. Of the 12 patients who received radiotherapy for GI SCC reported by Brenner et al., 11 out of 12 saw a partial/complete response or residual disease, with a survival of 3–17 months [6]. A larger 127 patient study of limited stage esophageal SCC demonstrated a 33 month median survival for patients who received chemoradiotherapy, while those who received surgery and chemotherapy had a 17.5 month median survival [26].

We looked to the literature for rectal SCC case reports, and found a broad range of treatment strategies and patient outcomes **(**Table 3**)**. Although some rectal SCC cases were treated with chemotherapy regimens similar to that of lung SCC, only a small portion of reported cases fell into that category. The most commonly employed treatment appeared to be surgery alone, followed by surgery and chemotherapy and lastly chemoradiotherapy. There were only three reported cases in which trimodality therapy was used. In some cases, radiotherapy was used for palliative measures in the setting of metastatic disease. The overall survival times in these case studies varied widely, and this may be partly due to differences in tumor stage at diagnosis. Although this literature search gives us a snapshot of the variety of treatment strategies on a case-by-case basis, it lacks the statistical power to guide treatment for future cases.

**Table 3:** **Summary of recent rectal small cell carcinoma case reports**

**Age**	**Sex**	**Chemotherapy**	**Radiation**	**Surgery**	**Metastasis**	**Outcome**	**Reference**
29	F	ETP+CDDP	RT (60Gy)	tumor resection (unspecified)	none	>42 mo	[29]
34	M	ETP+CBDCA	RT (unspecified)	proctocolectomy	none	>18 mo	[30]
68	F	ETP+CDDP	RT (39.6Gy)	abdominoperineal resection	liver	4 mo	[31]
51	M	CPM+DXR+vincristine+CBDCA+ETP	RT (30Gy)	-	none	>72 mo	[17]
48	M	ETP+CBDCA, CPM+DXR+vincristine	RT (pallative, craniospinal)	-	CNS and liver	17 mo	[19]
58	M	ETP+CDDP	RT (pallative, cranial 50.4Gy)	-	CNS and liver	3 mo	[32]
62	M	ETP+CDDP	RT (50Gy/2Gy fractions)	-	liver	12 mo	[33]
45	F	-	RT (pallative)	-	bone and liver	3 mo	[34]
68	M	-	RT (unspecified)	-	unknown	12 mo	[35]
83	M	-	RT (unspecified)	-	none	>3 mo	[35]
68	F	chemotherapy (unspecified)	-	-	liver, lymph nodes	>4.5 mo	[35]
46	F	chemotherapy (unspecified)	-	colectomy	liver, lymph nodes	3 mo	[35]
40	F	ETP+CPM+DXR	-	lower anterior resection	CNS	6 mo	[20]
69	M	DXR, 5-FU+CDDP, ETP+CDDP	-	rectal amputation, lymphadenectomy	unknown	>16 mo	[36]
36	M	CDDP+irinotecan	-	proctocolectomy	lymph nodes	>8 mo	[37]
46	M	ETP+CDDP	-	liver resection and abdominoperineal resection	liver	>48 mo	[38]
74	M	FOLFOX+bevacizumab	-	rectosigmoidectomy	liver	3 mo	[39]
46	M	5-FU+CDDP	-	abdominoperineal resection, lymphadenectomy	liver, lymph nodes, bone	8 mo	[18]
83	F	-	-	colectomy	liver, lymph nodes	>26 mo	[35]
50	F	-	-	radical resection of tumor	unknown	8 mo	[40]
78	M	-	-	endoscopic submucosal dissection	lymph nodes	6 mo	[41]
47	M	-	-	rectal amputation, lymphadenectomy	lymph nodes	>37 mo	[42]
63	M		-	tumor resection (unspecified)	liver, lymph nodes	10 mo	[43]
39	F	-	-	local resection, radical resection, lymphadenectomy	local lymphatic spread	>84 mo	[44]
46	F	-	-	diverting colostomy	liver, lymph nodes	unknown	[21]
74	M	-	-	tumor resection (unspecified)		2 mo	[21]
80	F	-	-	colectomy	none	unknown	[35]
34	F	-	-	colectomy	none	>6 mo	[35]
74	F	-	-	colectomy	liver, lymph nodes	3 mo	[35]

Age, sex, treatment, metastasis and outcome from rectal SCC cases found in the literature. Abbreviations: RT, radiation therapy. Gy, gray. DXR, doxorubicin. 5-FU, 5-fluorouracil. CDDP, cisplatin. CMP, cyclophosphamide. ETP, etoposide. CBDCA, carboplatin. FOLFOX, folinic acid, 5-FU and oxaliplatin chemotherapy regiment. CNS, central nervous system.

We cannot rule out the benefit of surgery from this study due to small sample sizes and insufficient power. It is also important to consider the limitations of using this retrospective SEER analysis to draw strong correlative conclusions. There is no chemotherapy data available in our SEER cohort, so we cannot account for any effects arising from differences in chemotherapy regimens. We were not able to determine from the SEER database whether radiation given to the RT group was with curative or palliative intent. Within our No_RT arm, a significantly greater proportion of the patients had a history of other prior malignancy before receiving a diagnosis of rectal SCC; these patients may have received previous radiation treatment to the pelvic area or previous chemotherapy. Despite the inherent limitations with using the SEER database, this study provides evidence for the benefit of radiation therapy for the treatment of rectal SCC.

## Conclusions

Given the scarcity of literature, we performed an analysis of rectal SCC patients entered into the SEER database from 1988 to 2010 in the United States. We were interested in the effect of radiation therapy and surgery on overall survival in rectal SCC. Radiation has been shown to confer a benefit in other extrapulmonary SCCs and in lung SCC [6,9,13-16,26]. And, the role of surgery in extrapulmonary SCC is controversial [2,9,13,23,24]. Our analysis revealed a significant survival benefit to patients that received radiotherapy, with or without surgery. Radiation therapy was the strongest prognostic variable amongst sex, age at diagnosis or surgery for overall survival. Although this SEER analysis has limitations, such as lack of chemotherapy information and retrospective study design, it provides guidance on how to manage this rare and aggressive cancer. These findings may also influence future prospective studies to establish a standard treatment regimen for rectal SCC.
